# Target Modulation by a Kinase Inhibitor Engineered to Induce a Tandem Blockade of the Epidermal Growth Factor Receptor (EGFR) and c-Src: The Concept of Type III Combi-Targeting

**DOI:** 10.1371/journal.pone.0117215

**Published:** 2015-02-06

**Authors:** Suman Rao, Anne-Laure Larroque-Lombard, Lisa Peyrard, Cédric Thauvin, Zakaria Rachid, Christopher Williams, Bertrand J. Jean-Claude

**Affiliations:** 1 Cancer Drug Research Laboratory, Department of Medicine, Division of Medical Oncology, McGill University Health Center/Royal Victoria Hospital, 687 Pine Avenue West Rm M7.19, Montreal, Quebec, H3A 1A1 Canada; 2 Chemical Computing Group Inc., 1010 Sherbooke St. West, Suite #910, Montreal, QC, H3A 2R7 Canada; Wayne State University, UNITED STATES

## Abstract

Cancer cells are characterized by a complex network of interrelated and compensatory signaling driven by multiple kinases that reduce their sensitivity to targeted therapy. Therefore, strategies directed at inhibiting two or more kinases are required to robustly block the growth of refractory tumour cells. Here we report on a novel strategy to promote sustained inhibition of two oncogenic kinases (Kin-1 and Kin-2) by designing a molecule K1-K2, termed “combi-molecule”, to induce a tandem blockade of Kin-1 and Kin-2, as an intact structure and to be further hydrolyzed to two inhibitors K1 and K2 directed at Kin-1 and Kin-2, respectively. We chose to target EGFR (Kin-1) and c-Src (Kin-2), two tyrosine kinases known to synergize to promote tumour growth and progression. Variation of K1-K2 linkers led to AL776, our first optimized EGFR-c-Src targeting prototype. Here we showed that: (a) AL776 blocked EGFR and c-Src as an intact structure using an *in vitro* kinase assay (IC50 EGFR = 0.12 μM and IC50 c-Src = 3 nM), (b) it could release K1 (AL621, a nanomolar EGFR inhibitor) and K2 (dasatinib, a clinically approved Abl/c-Src inhibitor) by hydrolytic cleavage both *in vitro* and *in vivo*, (c) it could robustly inhibit phosphorylation of EGFR and c-Src (0.25–1 μM) in cells, (d) it induced 2–4 fold stronger growth inhibition than gefitinib or dasatinib and apoptosis at concentrations as low as 1 μM, and, (e) blocked motility and invasion at sub-micromolar doses in the highly invasive 4T1 and MDA-MB-231 cells. Despite its size (MW = 1032), AL776 blocked phosphorylation of EGFR and c-Src in 4T1 tumours *in vivo*. We now term this new targeting model consisting of designing a kinase inhibitor K1-K2 to target Kin-1 and Kin-2, and to further release two inhibitors K1 and K2 of the latter kinases, “type III combi-targeting”.

## Introduction

**Fig 1:**
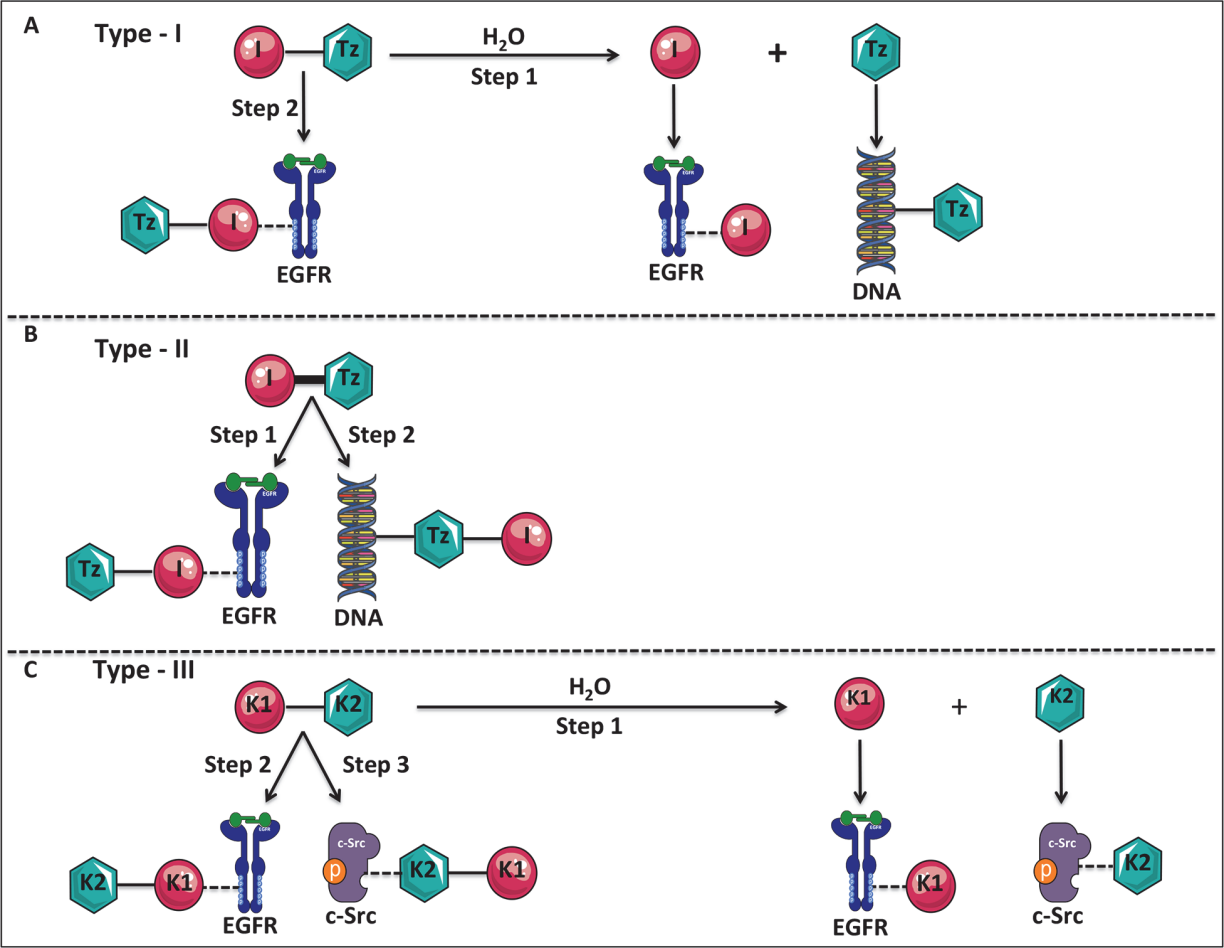
Prototypes of type-I, type-II and the novel type-III targeting molecules. (**A**) The type-I molecule (I-Tz) was designed to contain an EGFR tyrosine kinase inhibitor (I) and a DNA alkylating triazene (Tz) moiety bridged by a hydrolysable linker. The type-I molecule can inhibit EGFR as an intact structure (step 2), or upon undergoing hydrolysis to release the two moieties (I + Tz), but the molecule is only capable of targeting DNA through the release of its Tz moiety (step 1). (**B**) The type-II molecule was designed to contain the EGFR tyrosine kinase inhibitor (I) and the DNA alkylating triazene moiety (Tz) connected via a non-hydrolysable linker. This type-II molecule is capable of targeting both EGFR and DNA as an intact structure through each of its targeting arm (steps 1 and 2). (**C**) The novel type-III molecule (K1-K2) is designed to contain two tyrosine kinase inhibitors connected via a hydrolysable linker where K1 is targeted to Kin-1 (EGFR) and K2 to Kin-2 (c-Src). This molecule is “programmed” to exhibit both type-I and type-II like properties by inhibiting its two targets both as an intact structure (steps 2, 3) as well as upon undergoing hydrolysis to release inhibitors of EGFR and c-Src (step 1).

Current trend in cancer drug discovery is towards the design of multi-targeted agents [1]. This trend is driven by the observation that the attrition rates in the development of multi-targeted agents are significantly lower than that of single-targeted agents [2]. Indeed analysis of 974 anticancer agents from 1995 to 2007, in developmental phases (phase I to registration) led to an overall attrition rate of 82%. However, this rate fell to only 52% when the analysis was restricted to a subset of multi-targeted kinase inhibitors [2]. The clinical efficacy of multi-targeted agents is partly imputed to their ability to induce a tandem blockade of multiple targets that drive tumour progression and resistance to apoptosis in refractory tumours. However, despite the acknowledged potency of multi-targeted drugs, their rational design to inhibit specific oncogenic targets remains a tremendous challenge [3]. In the past, in the context of a novel multi-targeted approach termed “combi-targeting”, we designed inhibitors termed “combi-molecules” that can block targets as divergent as tyrosine kinase receptors and genomic DNA. We demonstrated their ability to kill tumour cells by blocking receptor phosphorylation, damaging DNA and down-regulating DNA repair proteins [4,5]. We classified such molecules as type I (i.e., those that require hydrolysis to fully exhibit their dual potency) and type II (i.e., those that could induce DNA damage and a tandem blockade of receptor mediated signaling without requirement for hydrolysis). As depicted in Fig. 1A, the type I molecule I-Tz was designed to release an EGFR tyrosine kinase inhibitor (I) and a DNA damaging species Tz (step 1). I-Tz was also designed to interact with EGFR as an intact structure (step 2) [6–8]. Conversely, I-Tz in its type II form is designed to inhibit EGFR tyrosine kinase and damage DNA without requirement for hydrolysis (Fig. 1B, steps 1 and 2) [9,10]. While this classification includes several types of agents directed at the epidermal growth factor receptor (EGFR) and DNA, the demonstration of the approach with two different tyrosine kinase targets remained a challenge [11–13]. Here, we designed a rational approach to give rise to a novel type of chimeric kinase inhibitor, that reconciles the type I and II targeting models. As shown in Fig. 1C (step 1), to target a cell expressing kinase 1 (Kin-1) and kinase 2 (Kin-2), we wish to design the molecule K1-K2 to behave like a type I targeted molecule, by conferring it a hydrolysable linker, which upon hydrolysis will release free K1 and K2 (inhibitors of kinases 1 and 2 respectively). In addition, the molecule is designed to possess an intrinsic dual K1/K2 targeting property as an intact molecule, thereby behaving as a type II molecule (Fig. 1C, steps 2 and 3). The expected advantage of the latter property lies in the fact that in the event that the hydrolysis of K1-K2 is slow inside the tumour cell, the intact structure can still induce a tandem blockade of the oncogenic targets Kin-1 and Kin-2. Overall, this novel targeting approach, which is now designated as type III combi-targeting, is designed to induce multispecies dynamic inside the cells with the dual tyrosine kinase Kin-1 and Kin-2 inhibition as a constant. Here we challenge this concept using an optimized molecule AL776, which was designed to block a receptor tyrosine kinase, EGFR, as Kin-1, and a non-receptor tyrosine kinase, c-Src, as Kin-2.

EGFR (Kin-1) and its family members are often overexpressed in many solid tumours including breast, lung, head and neck, prostate and colon and are associated with aggressive tumour progression and poor prognosis [14,15]. EGFR belongs to the HER family of receptor tyrosine kinases (RTKs) that consists of EGFR (HER1), HER2, HER3 and HER4. Upon ligand binding (e.g. EGF, TGF-α), the receptor undergoes homo/hetero-dimerization with its family members (or other RTKs) and activates several downstream signaling pathways including Ras-Raf-MAPK, PI3K/Akt and the signal transducer and activators of transcription (STAT), that are known to drive tumour growth, proliferation, survival and angiogenesis [16,17].

c-Src, our secondary target (Kin-2), is another important signaling protein activated downstream of EGFR. It is overexpressed in many solid tumours and is implicated in their growth, progression and metastasis [18]. Growth factor receptors other than EGFR including c-Met, PDGFR, IGF-1 receptor and several other membrane proteins including integrins, GPCRs, cytokine receptors are known to activate c-Src [18]. It has been demonstrated that tumours overexpressing both EGFR and c-Src have increased EGF-mediated DNA synthesis, soft agar growth, increased phosphorylation of receptor-binding proteins (Shc, PLC-γ) and increased tumourigenesis in nude mice [19]. Furthermore, it has also been shown that c-Src is responsible for the phosphorylation of a novel tyrosine residue, Y845 (a non-autophosphorylation site in the activation lip of the kinase domain) on EGFR, which leads to enhanced EGF-mediated DNA synthesis [20]. In addition, previous work by Bao *et al*. [21] demonstrated that c-Src prevents c-Cbl mediated endocytosis and ubiquitination of EGFR, thereby prolonging its signaling at the cell surface. Overall, EGFR and c-Src synergize to promote tumour growth and progression [20,22], and as a result, these deleterious interactions between the two kinases represent an appropriate multi-signaling context to challenge the new type III combi-targeting model.

Here we describe the synthesis, multispecies dynamics and the mechanism of action of the optimized prototype K1-K2 targeting molecule, AL776. We also report on its ability to modulate the two targets EGFR (Kin-1) and c-Src (Kin-2) *in vivo*.

## Results

### Design and kinase inhibition by K1-K2 molecular prototypes

**Fig 2:**
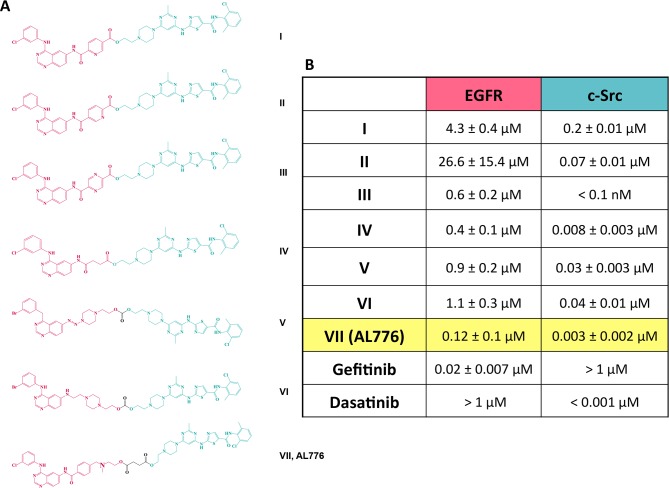
Series of EGFR-c-Src targeting type III molecules and their kinase inhibitory potency *in vitro*. (**A**) EGFR-c-Src targeting type III molecules were designed and synthesized in our laboratory using quinazoline moieties (red) as the EGFR targeting head and dasatinib as the c-Src inhibitory arm (green), connected through different hydrolysable linkers. (**B**) *In vitro* kinase assay was used to determine the potency of each molecule in the series to competitively bind and inhibit the ATP binding pocket of the tyrosine kinase domains of EGFR and c-Src. Gefitinib and dasatinib were used as control drugs for comparison, and the IC_50_ values of kinase inhibition were determined using the GraphPad Prism 6.0 software. Each value represents the average IC_50_ from three independent experiments, carried out in duplicate.

A series of K1-K2 molecules designed and synthesized in our laboratory is presented in Fig. 2A. The quinazoline backbone being highly tolerant of bulky substituents at the 6-position [23], was chosen as the EGFR targeting scaffold. This scaffold is common to many clinical inhibitors of EGFR including gefitinib, erlotinib, lapatinib and afatinib and is known to anchor into the ATP binding site of the receptor [24]. In the past, we found the pyrazolo pyrimidine class of inhibitors of c-Src to be extremely sensitive to substituent changes [11]. Therefore, we selected the thiazolylaminopyrimidine backbone of dasatinib, a clinically approved and potent inhibitor of Abl and c-Src, as the c-Src targeting scaffold [25]. To facilitate intracellular hydrolysis, we selected ester- or carbonate-based linkers between K1 and K2. This led to the structures shown in Fig. 2A, with IC_50_ values for EGFR and c-Src inhibition measured by an *in vitro* kinase assay. Of all the linkers studied, the succinic acid one led to the most potent dual EGFR-c-Src targeting molecule. The latter, AL776 showed an IC_50_ of 0.12 μM for EGFR kinase inhibition and 3 nM for c-Src kinase inhibition (Fig. 2B). Therefore, AL776 was selected as our K1-K2 prototype in the study.

### Synthesis of AL776

**Fig 3:**
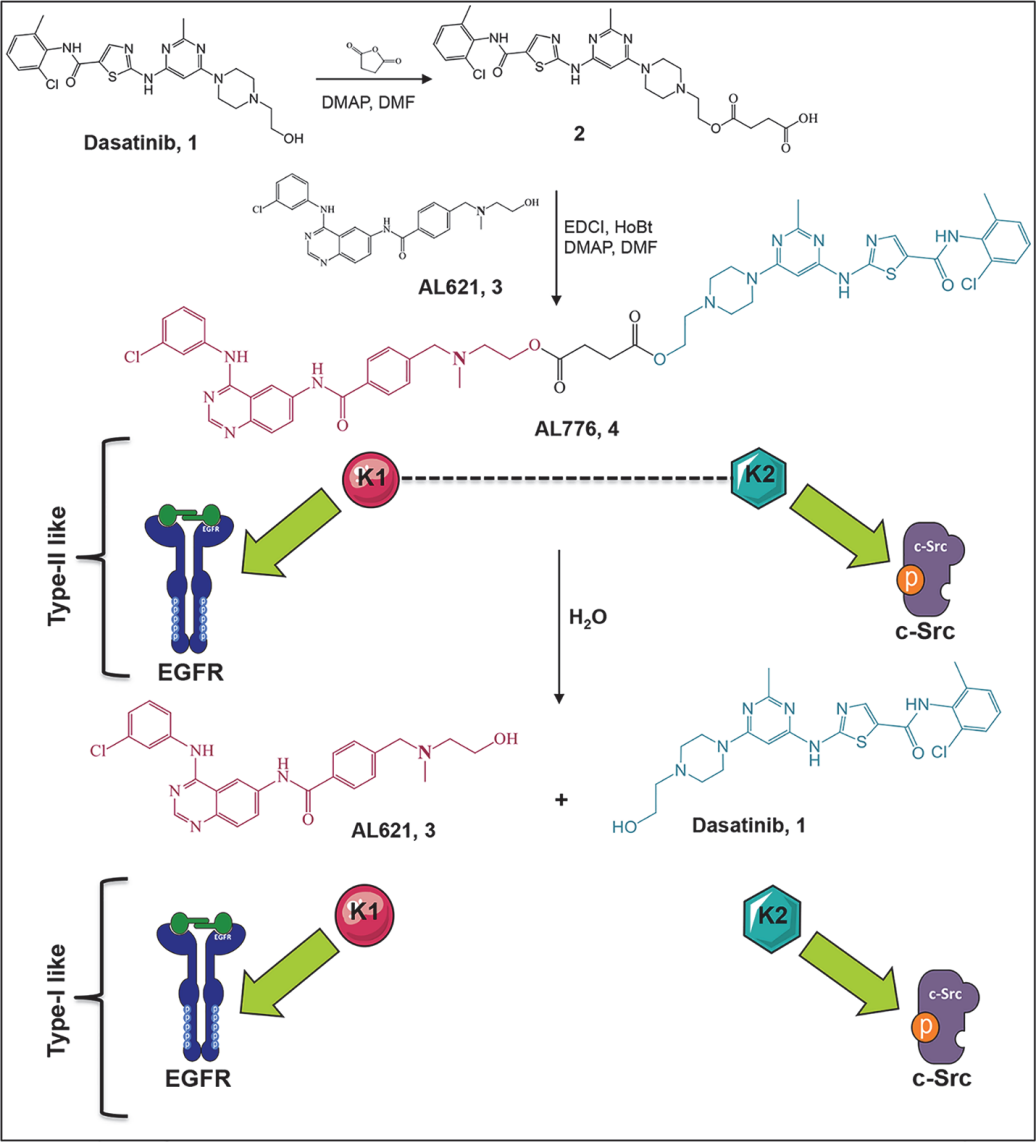
Synthesis and hydrolysis of AL776, the lead K1-K2 prototype targeting EGFR and c-Src. The synthesis of AL776 was carried out in our laboratory according to the steps indicated above. The resulting type III K1-K2 molecule is designed to undergo hydrolysis inside the cells and release a potent EGFR tyrosine kinase inhibitor (K1) termed AL621 and a potent c-Src tyrosine kinase inhibitor (K2) dasatinib (type I). AL776 is also capable of exerting its dual inhibitory property by directly interacting with each target as an intact molecule (type II).

The synthesis of AL776 proceeded according to Fig. 3. Dasatinib was treated with an excess of succinic anhydride to give compound **1**, which was coupled with AL621 (a potent EGFR tyrosine kinase inhibitor with IC_50_ = 3 nM [12]) in the presence of EDCI, HOBt and DMAP to give **VII** (AL776) as an analytically pure white powder following purification by preparative TLC. We predicted that the hydrolysis of AL776 would restore its primary synthetic elements (i.e. AL621 as K1 and dasatinib as K2).

### Kinetics of hydrolysis of AL776 *in vitro* and *in vivo*

**Fig 4:**
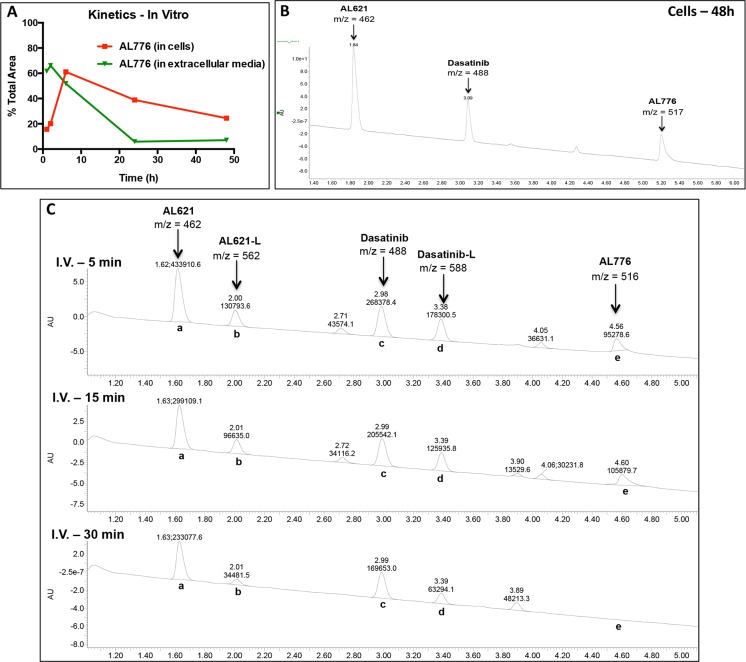
*In vitro* and *in vivo* hydrolysis of AL776 using high performance liquid chromatography (HPLC) and mass spectrometry (MS) analyses. (**A**) The kinetics of entry into the cells and degradation of AL776 inside the cells were monitored using HPLC analysis. NIH3T3-Her14 (EGFR transfected) cells were treated with 25 μM of AL776 for 1h, 2h, 6h, 24h and 48h, after which the cells and the corresponding extracellular media were collected and processed according to the procedure described in the Materials and Method section. The area under the curve (AUC) for the AL776 peak was determined and its percentage compared with all the other peaks was calculated and plotted. (**B**) A representative spectrum obtained from liquid chromatography (LC)-mass spectrometry (MS) analysis in cells treated with AL776 for 48h is shown with m/z = 462 (AL621), m/z = 488 (dasatinib) and m/2z = 517 (AL776). (**C**) The kinetics of AL776 hydrolysis in the plasma of CD-1 mice injected with 80 mg/kg of the drug was monitored 5, 15 and 30 min post-administration. LC-MS chromatograms at different time points with m/z values for intact AL776 and its metabolites are shown: m/z = 462 for AL621, m/z = 562 for AL621-L (succinic acid linked-AL621), m/z = 488 for dasatinib, m/z = 588 for dasatinib-L (succinic acid linked-dasatinib), m/2z = 516 for AL776.

The kinetics of hydrolysis of AL776 was studied both *in vitro* using NIH3T3-Her14 (EGFR transfected) cells and *in vivo* in CD-1 mice following i.p. and i.v. injection. *In vitro*, high performance liquid chromatography (HPLC) analysis of the extracellular medium and isolated whole cells revealed that AL776 was stable enough to slowly diffuse into the cells with minimal extracellular decomposition. As shown in Fig. 4A, 24–48h later, AL776 was detectable inside the cells but not in the extracellular medium, indicating that the absorption equilibrium was shifted towards intracellular retention of the molecule. AL776 slowly degraded inside the cells and liquid chromatography-mass spectrometry (LC-MS) analysis confirmed that the two released metabolites were AL621 and dasatinib (Fig. 4B). The observation of detectable levels of AL776 as long as 48h after treatment indicates that, as predicted, in addition to the individual metabolites K1 and K2 released inside the cells, intact K1-K2 may also contribute to their response to drug treatment. A representative spectrum is shown in Fig. 4B with m/z corresponding to the major metabolites along with intact AL776.

**Fig 5:**
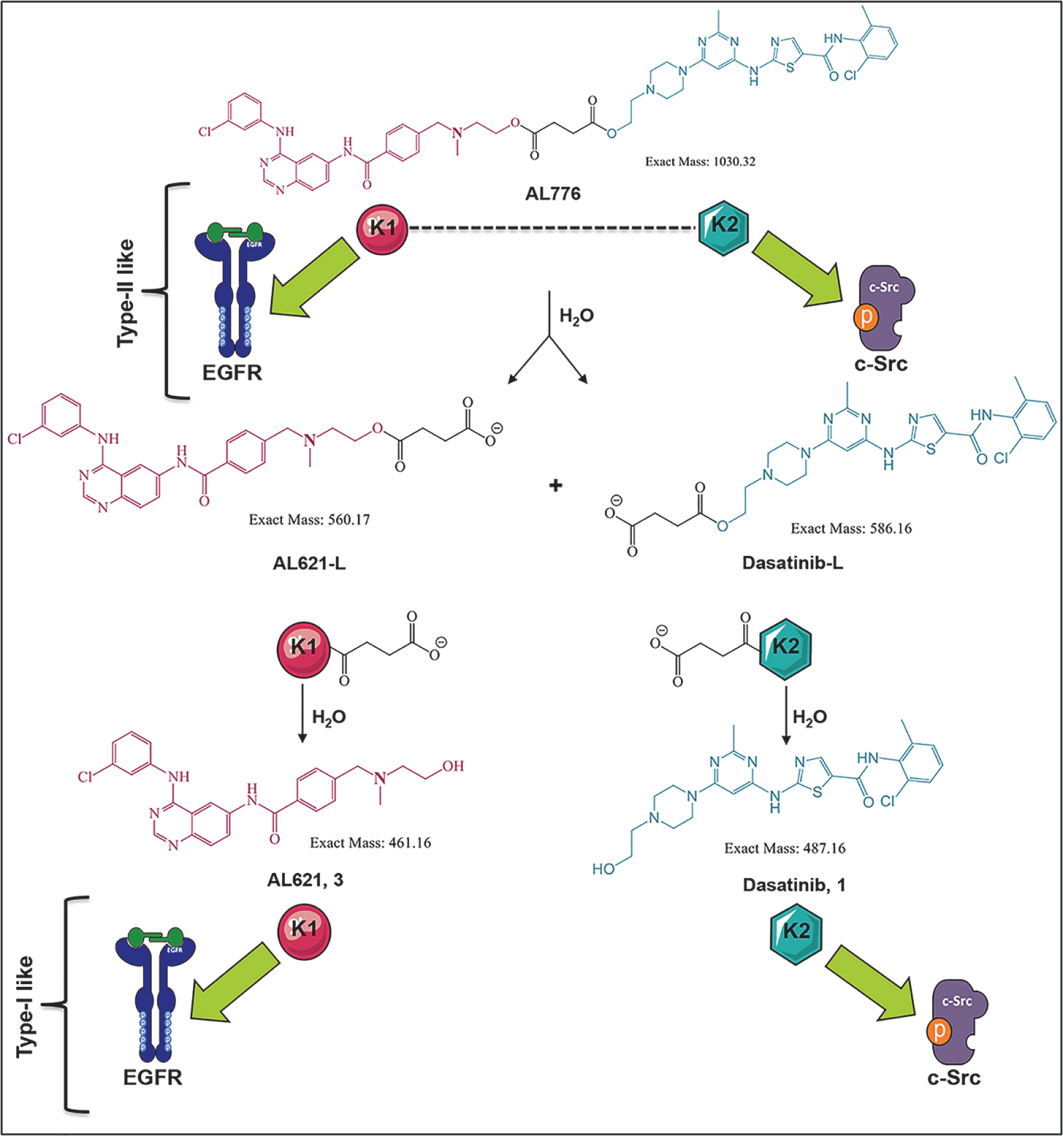
Schematic representation of AL776 hydrolysis and its hydrolyzed metabolites. Based on LC-MS characterization of the metabolites of AL776 both *in vitro* and *in vivo*, the hydrolytic scheme of AL776 is modified to represent all the major metabolites. The K1-K2 molecule undergoes hydrolysis to generate the acidic forms of AL621 and dasatinib (AL621-L and dasatinib-L), which are further metabolized to AL621 (K1 or EGFR inhibitor) and dasatinib (K2 or c-Src inhibitor).

Having studied the hydrolysis of AL776 *in vitro*, we sought to determine whether its degradation profile *in vivo* would parallel that *in vitro*. Mice were injected i.v. and i.p. with 80 mg/kg of AL776 and plasma collected at early time points. The results showed following i.p. injection, only the major metabolites were detected with no intact AL776 found in plasma. Therefore, the study was focused on i.v. injection where intact AL776 could be tracked at the earlier time points. Following i.v. injection, AL776 rapidly disappeared from the plasma with barely detectable levels as early as 30 min post-administration. However, after 30 min (see Fig. 4C), abundant levels of its two major metabolites (AL621 and dasatinib) were detected (Fig. 4C). Furthermore, LC-MS analysis showed that AL776 was not only cleaved to release AL621 (K1) + dasatinib (K2) but also generated metabolites carrying a succinic acid moiety, which we refer to as AL621-L (K1-L) and dasatinib-L (K2-L). Thus, as predicted, the major products of hydrolysis of AL776 were AL621 and dasatinib, which could be detected both *in vitro* and *in vivo*. In addition, since the levels of acidic metabolites AL621-L and dasatinib-L decreased rapidly, we believe that they were either eliminated or eventually converted to AL621 and dasatinib as proposed in Fig. 5.

### Molecular modeling and mode of binding of AL776 in the EGFR and c-Src kinase pocket

**Fig 6:**
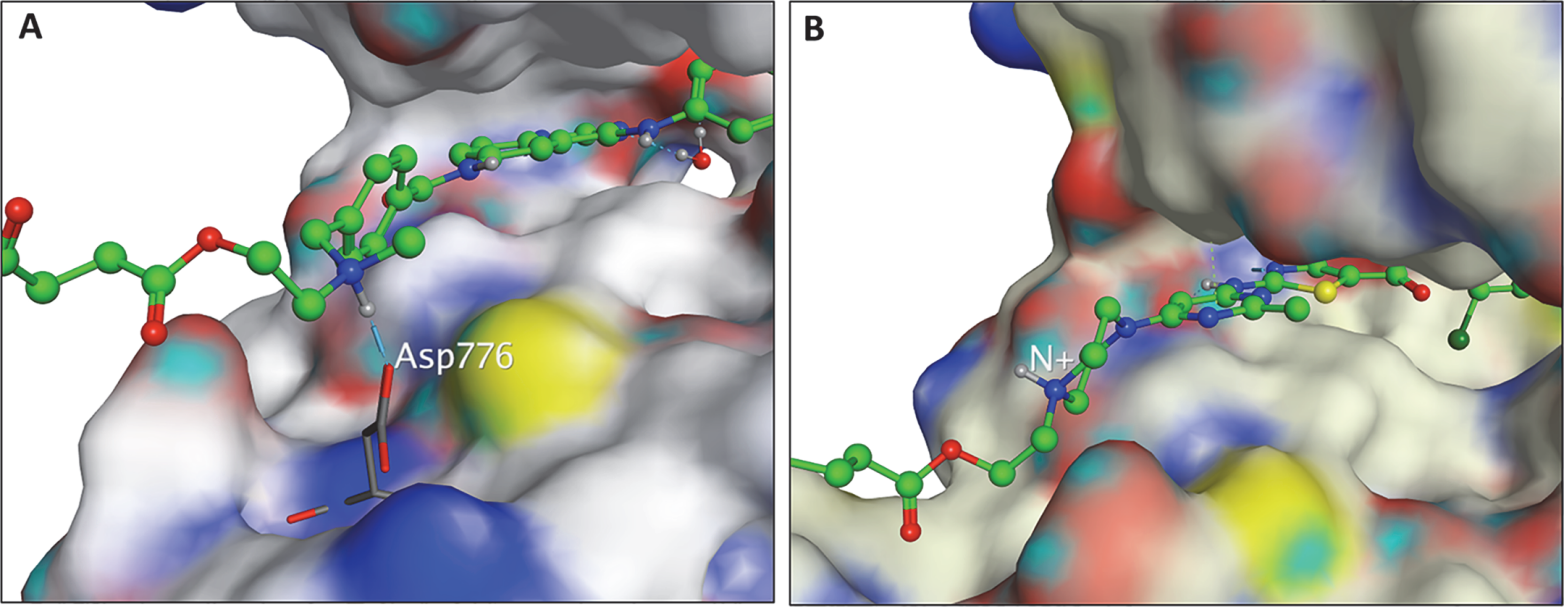
Molecular modeling of AL776. (**A**) AL776 modeled in the EGFR kinase-binding pocket using the Protein Data Bank (PDB) with code 1M17. The quinazoline moiety can bind to the hinge region in a manner analogous to erlotinib, while the linker-dasatinib portion of AL776, exposed to solvent, can adopt a number of conformations. The protonated form of the tertiary amine in AL776 can interact with Asp776. (**B**) AL776 modeled in the c-Src pocket using the PDB with code 3G5D. The dasatinib moiety of AL776 binds to the hinge portion of the c-Src ATP binding pocket in a pose identical to dasatinib while the linker-quinazoline portion of AL776 points out into solvent and can adopt many conformations.

One of the primary requirements of the K1-K2 prototype is to possess strong inhibitory potency against Kin-1 and Kin-2, both as an intact molecule as well as upon undergoing hydrolysis to release K1 directed at Kin-1 and K2 at Kin-2. Having found that AL776 (K1-K2) in an *in vitro* kinase assay possessed dual EGFR and c-Src targeting property as an intact structure, it was important to determine how it could probably bind to the EGFR and c-Src kinase domain. Thus, molecular modeling was used to map the binding of the intact structure to EGFR or c-Src. AL776 was modeled in the EGFR kinase pocket using the 1M17 Protein Data Bank (PDB) structure as a starting point. The quinazoline portion of bound erlotinib [26] in 1M17 was used as a template to construct and minimize a bound pose of AL776. Despite the large size of AL776, the quinazoline moiety could bind to the 1M17 structure in a pose analogous to erlotinib. In this pose the linker-dasatinib portion of AL776 points out of the ATP binding pocket towards solvent, allowing for conformational flexibility. Furthermore, the tertiary alkyl nitrogen atom of AL776 is in a position such that the protonated form can interact via a hydrogen-bond/ionic interaction with the carboxylate group of Asp776. A sample pose of AL776 showing the N+-Asp776 interaction is given in Fig. 6A.

AL776 was also modeled in the c-Src kinase pocket using the PDB structure 3G5D [27], It was constructed and minimized in 3G5D starting with the bound dasatinib ligand as the template. The dasatinib portion of AL776 is in the same position as dasatinib in 3G5D, and maintains the same protein-ligand non-bonded interactions as dasatinib. The linker-quinazoline portion of AL776 is solvent exposed and makes no specific interactions with the c-Src ATP-binding pocket. A conformational search performed on the linker-quinazoline portion of AL776 produced many diverse conformations, none of which shows any specific H-bond or electrostatic interaction between AL776 atoms and c-Src residues. Thus, when bound to c-Src, the dasatinib portion of AL776 can adopt a binding mode identical to that of dasatinib in 3G5D, while the linker-quinazoline portion of the AL776 is free to adopt a number of conformations, none of which appear particularly favored due to a specific interaction with residues at the mouth of the c-Src ATP binding pocket. A sample pose of AL776 modeled in 3G5D is given in Fig. 6B.

### Target modulation and effect on growth inhibition, survival and invasion in cells

(a) Downregulation of EGFR and c-Src phosphorylation by AL776

**Fig 7:**
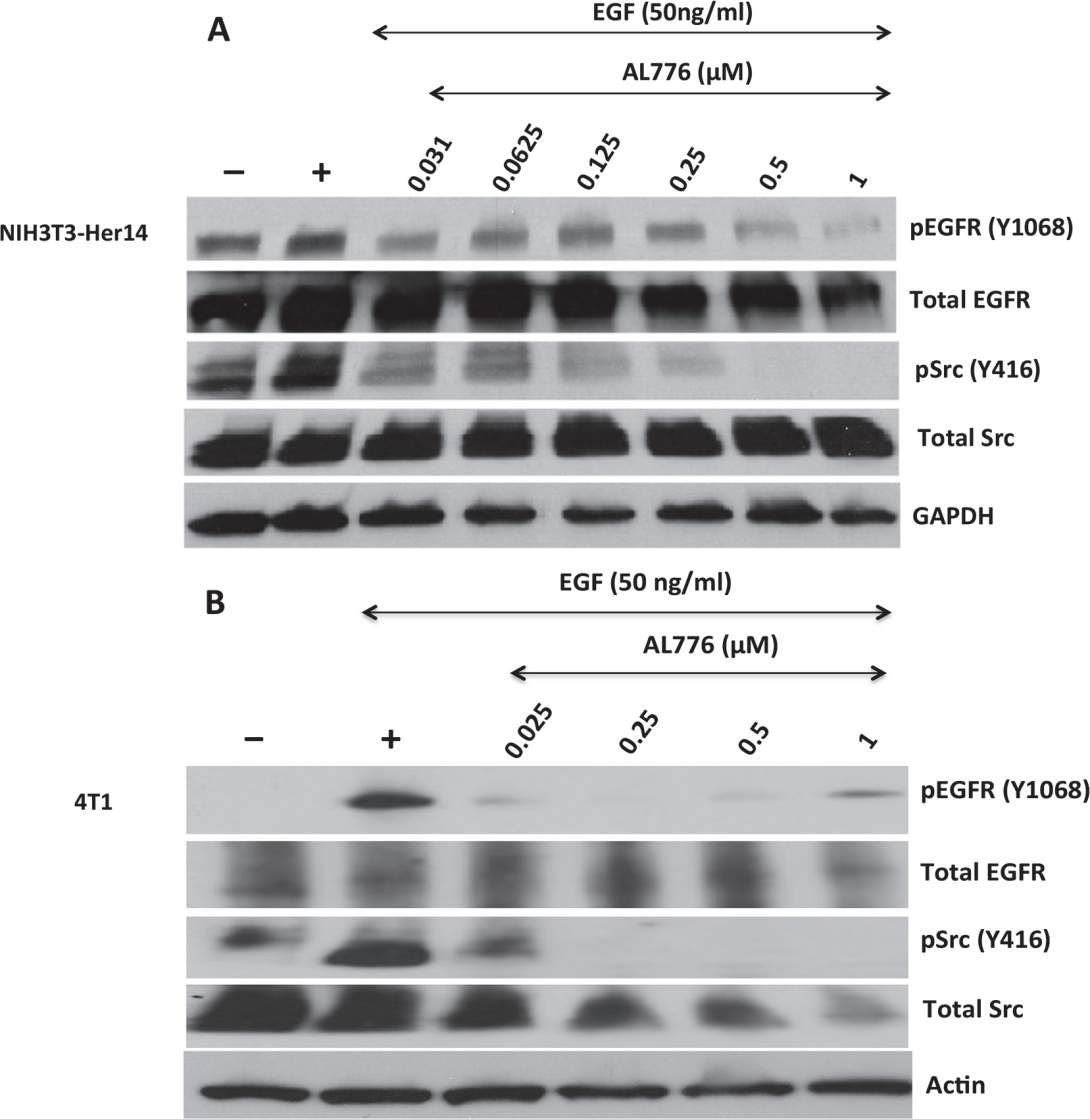
Target modulation using western blot analysis. (**A**) NIH3T3-Her14 (EGFR transfected cells) and (**B**) 4T1 mouse mammary tumour cells were starved using serum-free medium for 24h and treated with varying concentrations of AL776 for 2h. The drug was removed from the medium and the cells were stimulated with 50 ng/ml of EGF for 30 min. Cells were extracted, lysed and western blot analysis was carried out according to the protocol described in the Materials and Methods section. Membranes were probed with phospho-EGFR (Y1068), phospho-Src (Y416), total EGFR, Src and housekeeping (Actin or GAPDH) antibodies.

The contribution of the multiple species in the cells to inhibition of EGFR and c-Src phosphorylation was analyzed by immunoblot assay in NIH3T3-Her14 mouse fibroblast cells transfected with EGFR (Fig. 7A) and in the highly invasive 4T1 mammary tumour cells (Fig. 7B). Cells were treated with different concentrations of AL776 for two hours followed by stimulation with EGF (50 ng/ml) for 30 minutes. The results showed that AL776 induced a dose-dependent inhibition of both EGFR and c-Src phosphorylation with maximal inhibition at a concentration as low as 1 μM. The results obtained from the kinetics of hydrolysis of AL776 inside the cells after 2h are consistent with the presence of intact AL776 along with AL621 and dasatinib (S1 Fig.).

**Fig 8:**
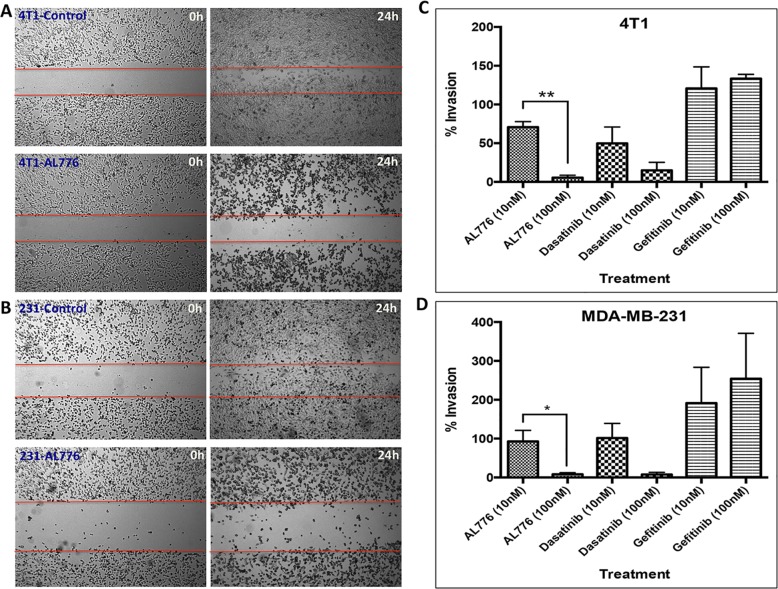
Anti-motility and anti-invasive properties of AL776 in 4T1 mouse mammary tumour and MDA-MB-231 triple negative breast cancer cell lines. (**A**) 4T1 and (**B**) MDA-MB-231 cells were treated with 100 nM of AL776 or control drugs gefitinib or dasatinib for a period of 24h and the wound-closure (scratch) was monitored at both 0h and 24h time points. (**C**) 4T1 or (**D**) MDA-MB-231 cells were treated with varying doses of AL776 (in comparison with dasatinib or gefitinib) for a period of 24h in a Boyden Chamber invasion assay. Cells were plated in serum-free media (top chamber) and allowed to invade across the layer of matrigel towards media containing 10% FBS (bottom chamber) through chemotaxis. Drugs were added to both the top and bottom chambers to maintain a uniform distribution. Cells were fixed, stained and quantified to generate the percentage of invading cells across the matrigel in comparison with untreated control cells. The histograms represent the average ± SEM of three independent experiments. Statistical analysis was performed using the unpaired two-tailed t-test and p values were obtained (p < 0.05 is significant): 4T1 ** (p = 0.001), MDA-MB-231 * (p < 0.05).

(b) Anti-motility and anti-invasive properties of AL776

c-Src being a key tyrosine kinase in the signaling pathways associated with motility and invasion, we thought it of interest to evaluate the effects of AL776 on motility and invasion using the wound-healing and the Boyden chamber assay respectively. These experiments were performed in the highly invasive 4T1 and MDA-MB-231 breast cancer cell lines. Both cell lines were used in these assays due to their high levels of c-Src expression, which is a key oncogene in driving tumour invasion and metastasis [28,29]. The assay was carried out by exposing the cells to the drug for 24h, a time point at which 50% of intact AL776 was found in the cells. Wound-healing assay results showed that AL776 at 0.1 μM blocked wound-closure after a 24h drug exposure in both cell lines (Fig. 8A and B). Boyden chamber invasion assay results showed that AL776, like dasatinib, strongly inhibited invasion at a concentration as low as 0.1 μM. However, gefitinib was mostly unable to block invasion at such low doses (Fig. 8C and D), indicating that c-Src and not EGFR is primarily responsible for the invasive properties of these cells.

(c) Growth inhibitory and apoptotic properties of AL776

One of the premises of designing multi-targeted molecules is to induce pleiotropic effects without losing selectivity for the primary targets. Having shown that AL776 was capable of blocking invasion, we tested the ability of its multi-targeted properties to translate into selective growth inhibition and apoptosis in different cell lines.

**Fig 9:**
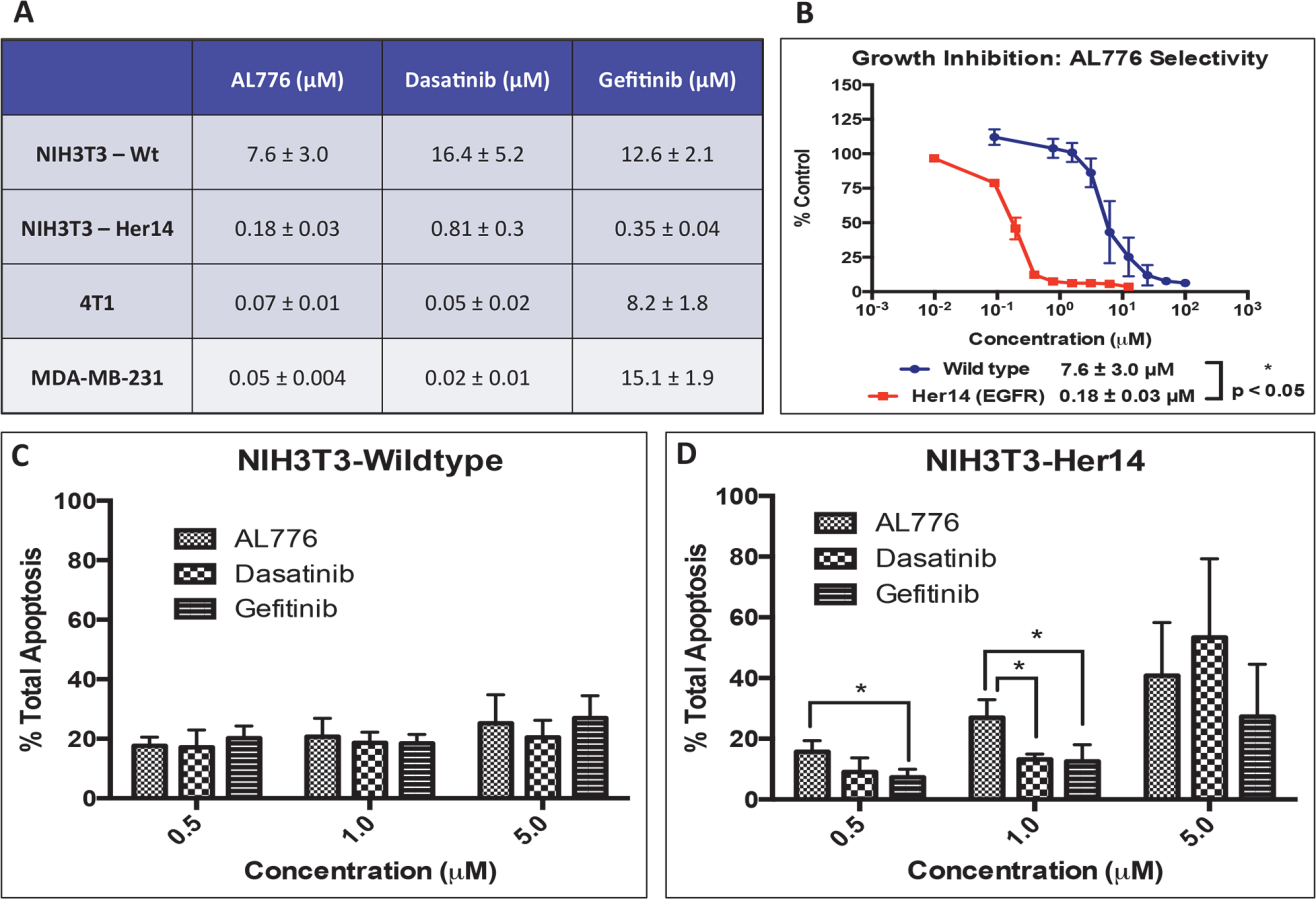
Growth inhibitory and apoptotic properties of AL776. (**A**) Growth inhibition was carried out in NIH3T3 wild type and Her14 (EGFR transfected), 4T1 and MDA-MB-231 cell lines using the sulforhodamine B (SRB) assay. Cells were treated with varying doses of AL776, gefitinib or dasatinib for a period of 5 days following which cells were fixed, stained and quantified. Each experiment was repeated at least four times and carried out in triplicates. (**B**) Comparison of the growth inhibition curves and corresponding IC_50_ values of AL776 in the isogenic NIH3T3 wild type and EGFR transfected cell lines. Each point represents the average ± SEM of five independent experiments carried out in triplicates. The difference between their average IC_50_ values was statistically significant with p < 0.05. (**C**, **D**) Annexin V and propidium iodide staining (PI) of cells was used to determine the percentage of apoptosis (early + late) induced by AL776, gefitinib and dasatinib. Cells were treated with different doses of AL776 for 48h, collected, stained and analyzed using flow cytometry. The histogram represents the average ± SEM of three independent experiments with p < 0.05 in the NIH3T3-EGFR transfected cell line.

Its growth inhibitory property was tested by treating the NIH3T3 wild type, Her14 (EGFR transfected), MDA-MB-231 and 4T1 cell lines with a dose range of AL776 or gefitinib or dasatinib for a period of 5 days and the IC_50_ values for growth inhibition were determined. The results showed that AL776 induced strong growth inhibition in NIH3T3-Her14 cells with an IC_50_ of 0.18 μM and showed 2–4 fold higher potency compared with clinical drugs gefitinib or dasatinib. In MDA-MB-231 and 4T1 cell lines, it induced strong growth inhibition, like dasatinib, with IC_50_ values in the sub-micromolar range (Fig. 9A). More importantly, AL776 was selectively more potent in NIH3T3 cells transfected to overexpress EGFR when compared with their wild type counterpart (Fig. 9B).

The ability of AL776 to induce apoptosis was assessed using NIH3T3 wild type and EGFR transfected cells treated with 0.5, 1 or 5 μM doses of AL776, gefitinib or dasatinib for a period of 48h and analyzed using flow cytometry. Total apoptosis induced in these cells was calculated as the sum of the percentage of early (annexin V staining) and late apoptosis (annexin V + PI staining). The results showed that while all three drugs were selectively cytotoxic towards EGFR transfected cells, AL776 induced a higher level of apoptosis than gefitinib or dasatinib at the 1μM dose (Fig. 9C, D). Interestingly, HPLC analysis showed that 48h after drug exposure, while a major part of AL776 had undergone hydrolysis to release its two inhibitory arms (AL621 and dasatinib), a small percentage of the intact molecule was still present inside the cells (Fig. 4B). This indicated that inhibition of EGFR and c-Src could not only be mediated by AL621 and dasatinib, but also by intact AL776.

### Target modulation *in vivo*

**Fig 10:**
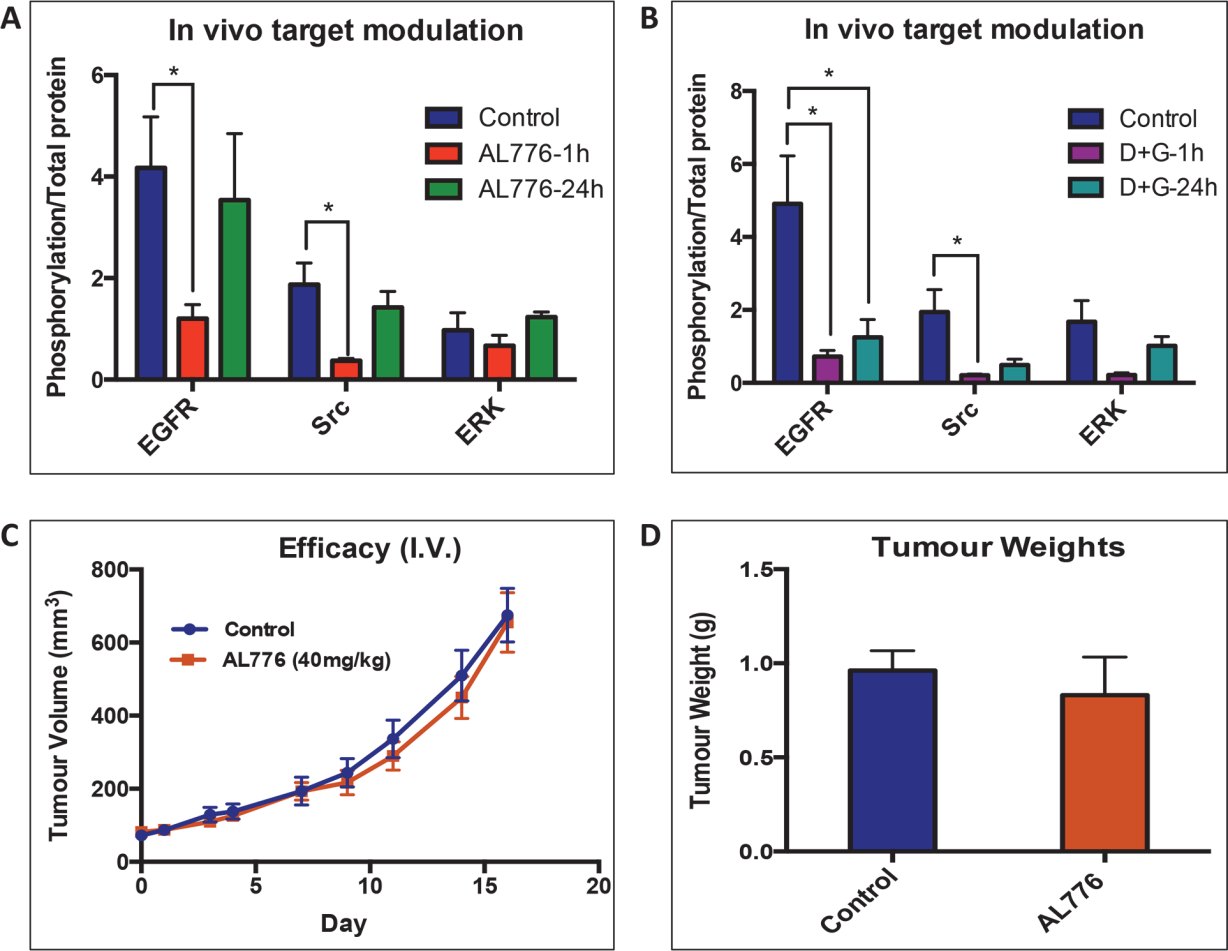
Pharmacodynamics in 4T1 tumours. (**A**, **B**) Female Balb/c mice (n = 4) bearing 4T1 tumours were treated with 40 mg/kg of AL776, 20 mg/kg each of gefitinib + dasatinib or vehicle and sacrificed 1h and 24h after drug administration (intravenous, i.v). Tumours were collected and western blot analysis was used to detect inhibition of phosphorylation of EGFR, c-Src and ERK1/2 by the drug, at different time points. The bands were quantified and represented as histograms and as a ratio of phospho-protein/total protein. Statistical significance was determined using multiple t-test and p < 0.05 was considered significant. (**C**) Efficacy study was carried out in female Balb/c mice (n = 6) bearing 4T1 tumours and treated with 40 mg/kg of AL776 or vehicle administered IV (once/day) for 5 consecutive days. Tumour growth was monitored for two weeks by measuring tumour volume on alternate days. Graphs representing the average tumour volumes ± SEM of the two groups are shown. (**D**) On the last day of the study, tumours were collected and their average ± SEM weight (g) from AL776 treated and the control groups (n = 6) are plotted as histograms.

Having shown that this new K1-K2 prototype could be hydrolyzed into the major bioactive species *in vivo* and to strongly block EGFR (Kin-1) and c-Src (Kin-2) phosphorylation *in vitro*, we sought to determine whether it could modulate its two targets in a tumour model *in vivo*. This was performed in comparison with the administration of the two free clinical inhibitors (gefitinib and dasatinib) targeting Kin-1 (EGFR) and Kin-2 (c-Src) using the mouse 4T1 cells in which we have shown the two targets to be modulated *in vitro* (Fig. 7B). The results showed that administration of AL776 (40 mg/kg) induced strong blockade of EGFR and c-Src phosphorylation *in vivo* 1h post-administration in a manner similar to the gefitinib + dasatinib combination. This was consistent with the observed inhibition of the two targets *in vitro*. Furthermore, *in vivo*, phosphorylation of the downstream phospho-protein ERK1/2 was strongly inhibited by AL776 and the 2-drug combination 1h post-administration. Inhibitions of phosphorylation of EGFR, c-Src and ERK1/2 by AL776 were reversed 24 h post-treatment but partially retained in mice treated with the two drug combinations (Fig. 10 A, B and S4 Fig.).

The ability of target modulation by AL776 to translate into inhibition of tumour growth was monitored by treating Balb/c mice bearing 4T1 tumours with 40 mg/kg of AL776 (i.v. injection) for 5 days. The results showed that AL776 was unable to block tumour growth (Fig. 10C) and animals treated with equivalent doses of gefitinib + dasatinib (given 20 mg/kg each, i.v.) experienced toxicities that precluded data collection. At the end of the study, tumour masses from control versus AL776 treated groups were weighed and there was no difference observed (Fig. 10D).

## Discussion

Over the past decade, while targeted therapy focused on single-drug-single-gene strategies, the field of polypharmacology emerged with a new paradigm shifting approach towards tumour targeting that consists of a one-drug-multiple gene approach [30,31]. Within the same context, we initiated a concept termed “combi-targeting” that sought to rationally design molecules to either degrade to generate multiple bioactive species (type I) or to hit two targets without any requirement for hydrolytic cleavage (type II). While we demonstrated the feasibility of these two approaches with molecular prototypes targeting DNA and EGFR [7,10], their application to the targeting of two different kinases (e.g. Kin-1 and Kin-2) remained a challenge. Molecular prototypes designed to induce a tandem inhibition of EGFR and c-Src lacked inhibitory potency against either kinase as an intact molecule [11,12]. Therefore it was not possible to prove the principle that consists of designing the K1-K2 molecule to hit the two targets without the requirement for hydrolysis and to be further hydrolyzed to release two potent inhibitors of Kin-1 and Kin-2 (see Fig. 1). Here, we sought to synthesize a potent dual EGFR-c-Src targeting prototype by introducing the thiazolylaminopyrimidine moiety as the c-Src tyrosine kinase inhibitor (TKI) while retaining the quinazoline head as the EGFR TKI and by altering the linkers (structures **I**-**VII**). It is important to mention here that the c-Src TKI dasatinib induces significant off-target inhibition of kinases including Bcr-Abl, members of the c-Src family of kinases and certain receptor tyrosine kinases including c-Kit, PDGFR-α, β and vascular endothelial growth factor receptor 2 [32,33] which may advantageously enhance the spectrum of potency of our combi-targeting compound in cells that harbor multiple compensatory pathways. Within the series of combi-targeting molecules (K1-K2) synthesized, we identified AL776 with IC_50_ values of 0.12 μM for EGFR and 3 nM for c-Src as an intact molecule, both IC_50_ concentrations being known to be associated with clinically active drugs [34,35]. Thus, AL776 was selected as a prototype to verify the type III combi-targeting postulates.

Hydrolysis studies have indeed shown that in cells, a fraction of the molecule remained intact (type II), as long as 48h post-treatment, while another portion was converted to EGFR and c-Src TKI (type I). These results indicate that indeed, up to 48h post-treatment, at least three major species were present, AL776 (K1-K2), AL621 (K1) and dasatinib (K2) in the cells. Evaluation of the biological impact of this multi-species milieu showed that a 2h drug exposure could induce both EGFR and c-Src blockade in whole cells. The presence of multiple hydrolytic products of AL776 in the cells indicate that dual inhibition of EGFR and c-Src in cells could primarily result from both type-I and type-II like mechanism, as highlighted in Fig. 1C. Evidence that the intact structure could induce strong EGFR and c-Src targeting potency in an ATP-competitive manner, is given by the enzyme assay in which the exposure time was only 8 minutes (assay time) at room temperature. Furthermore, molecular modeling confirmed the ability of AL776 to anchor in the ATP site of each kinase as an intact molecule. Also, the relative stability of AL776 in the intracellular milieu suggests that it can modulate its targets by a type II targeting mechanism (i.e. it does not require hydrolysis for generating its dual targeting properties). These results *in toto* confirmed AL776 as the first type III molecular probe of the combi-targeting postulates.

It is important to outline the fact that AL776, our K1-K2 prototype, could release the EGFR TKI AL621 (K1) with published IC_50_ value for EGFR kinase inhibition of 3 nM [*ca*. 40-fold more potent than the intact AL776 (K1-K2)] [12]. This property further strengthens the EGFR inhibitory potency of the parental K1-K2. Likewise, the potency of dasatinib (K2) remained in the nanomolar range (Fig. 2B). Thus, AL776 behaves like a prodrug of potent EGFR and c-Src inhibitors. Interestingly, analysis of the biological consequences of this property in isogenic cells expressing EGFR and c-Src (NIH3T3-EGFR transfected) showed that of all the binary targeting molecules tested, AL776 was the most potent on the EGFR transfectant (S2 Fig.), indicating high levels of EGFR selectivity. More importantly, the growth inhibitory potency of AL776 was 2–4 fold stronger than that of gefitinib (a nanomolar inhibitor of EGFR) and dasatinib (a low nanomolar inhibitor of c-Src). This superior effect of AL776, as evidenced by its ability to strongly block EGFR and c-Src phosphorylation in the cells, may be due to its ability to disrupt the synergistic interaction between EGFR and c-Src required to promote growth [19]. Interestingly, AL776 exhibited cytotoxic effects evidenced by its ability to induce high levels of apoptosis. Perhaps this may be secondary to the inhibition of EGFR and c-Src in the cells, two kinases that are known to activate the anti-apoptotic PI3K/Akt pathway [36–40].

EGFR and c-Src are known to promote motility and invasion in cells [41–43]. However our results showed that targeting c-Src alone with dasatinib was sufficient to induce anti-invasive and anti-motility effects in the same range as AL776. We believe that this is due to the fact that c-Src is the main driver of invasion in these cells, thereby leading to a non-consequential contribution of the strong EGFR inhibitory potency of AL776 and its released potent EGFR TKI, AL621. In corroboration, gefitinib alone showed 2-3-fold weaker anti-invasive effects than dasatinib.

Having proven the feasibility and potency of type III targeting *in vitro*, we subsequently tested our hypothesis *in vivo*. Interestingly, the metabolism of AL776 *in vivo* mimicked its intracellular degradation but with significantly faster kinetics and detectable levels of multiple species resulting from its partial hydrolysis or metabolism. The formation of the latter metabolites (AL621-L, Dasatinib-L and other unknown structures) may significantly enhance the multispecies dynamics *in vivo*. The rapid cleavage of AL776, which could only be detected at the early time points following i.v. injection may be mediated by the elevated levels of esterases known to be present in mouse plasma [44,45]. However it is important to note that following its complete disappearance (30 min), abundant levels of the two major metabolites (AL621 and dasatinib) were still present in the plasma. While the rapid degradation of AL776 *in vivo* does not support its ability to induce significant type II targeting in tumours *in vivo*, its dual targeting properties may still be supported by the major metabolites (e.g. AL621 and dasatinib) that it releases in the plasma. Indeed 1h after injection of AL776 both c-Src and EGFR phosphorylation were significantly inhibited in the tumours *in vivo* (p < 0.05) but these effects were reversed 24h post-injection and this did not translate into tumour growth inhibition. These results together with our *in vitro* data suggest that in order for type III targeting to be achievable *in vivo*, the rate of hydrolytic cleavage of AL776 must be slower. Further, structural modifications of the linker are required to achieve this goal.

**Fig 11:**
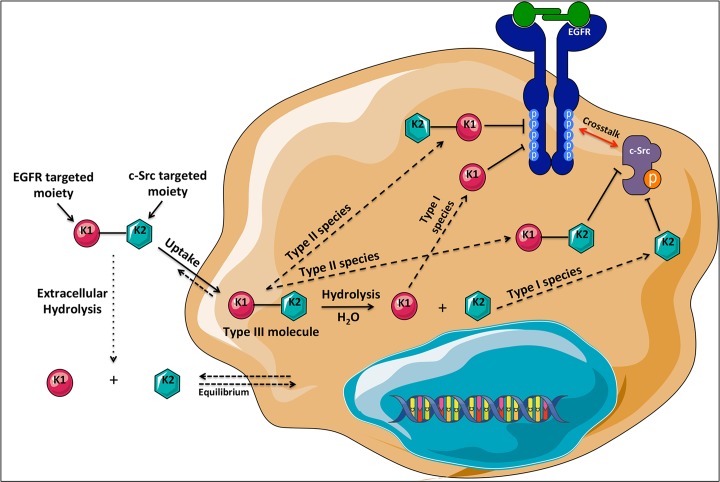
Schematic representation of type III combi-targeting mechanism with EGFR (K1) and c-Src (K2) as the kinase targets. Upon entering the cells, K1-K2 binds and inhibits its targets, EGFR and c-Src, both as an intact molecule and as a prodrug whose hydrolysis leads to the release of inhibitors of EGFR (K1) and c-Src (K2). The multiple bioactive species generated inside the cells inhibit both the targeted receptor and non-receptor tyrosine kinases, ultimately leading to inhibition of downstream signaling pathways associated with tumour growth and progression.

In summary, here we described the first prototype of multi-targeted molecule of type III and our results suggest that it was a suitable probe for the demonstration of this novel targeting mechanism *in vitro*. Briefly, as depicted in Fig. 11. K1-K2 penetrates the cells by passive diffusion where, through its ability to behave like a type II molecule, it can induce a tandem inhibition of EGFR and c-Src as an intact structure, or as a type I molecule whereby its intracellular hydrolysis leads to K1 targeted to EGFR and K2, targeted to c-Src. A fraction of K1-K2 could be hydrolyzed through a type I mechanism extracellularly, in which case, the released K1 and K2 could freely diffuse into the cells. Similarly, *in vivo*, the K1-K2 molecule and its metabolites were observed after i.v. injection. These multiple species are capable of inhibiting the two targets in a dose-dependent manner and modulate their phosphorylation status both *in vitro* and *in vivo*. Further work is required to ameliorate the *in vivo* potency of this novel type III combi-targeting approach.

## Conclusion

It is now increasingly recognized that the overall attrition rate in the development of multi-targeted kinase inhibitors is significantly low when compared with other types of drugs. This is believed to be due to the ability of such drugs to modulate multiple targets in the tumour cell. Here we demonstrated a novel approach to rationally design inhibitors to block two different kinases. We conclusively showed that a molecule could be designed as K1-K2 to block the two tyrosine kinases (Kin-1 and Kin-2) as an intact structure (K1-K2) and upon undergoing hydrolysis to release two potent inhibitors (K1 + K2) of Kin-1 and Kin-2, respectively. We showed that the two targets (EGFR and c-Src) were modulated *in vitro* and *in vivo* by our first prototype. While amenable *in vivo*, further work is required to overcome the bioavailability hurdles posed by the rapid hydrolysis of the resulting molecule. Our novel approach referred to as “type III combi-targeting” is the first targeting model to lead to a “prodrug-like” molecule with dual kinase activity, which is further “programmed” to generate even more potent inhibitors of these kinases (Kin-1 and Kin-2), upon hydrolysis.

## Materials and Methods

“Animal studies were carried out according to protocol # 4934 approved by the McGill Facility Animal Care Committee (FACC)”

### Chemistry

1H NMR spectra were recorded on a Varian 300 or 400 MHz spectrometer. Chemical shifts are given as δ values in parts per million (ppm) and are referenced to the residual solvent proton peak. Mass spectrometry was performed by the McGill University Mass spectroscopy Center and electrospray ionization (ESI) spectra were performed on a Finnigan LC QDUO spectrometer. Data are reported as m/z (intensity relative to base peak = 100). Elemental analyses were carried out by GCL & Chemisar Laboratories (Guelph, Ontario, Canada). All chemicals were purchased from Sigma-Aldrich.

Compound 1

To a solution of dasatinib (200 mg, 0.4 mmol) in dry dimethylformamide (DMF) (10 mL) at room temperature, an excess of succinic anhydride (41 mg, 4 mol, 10 eq.) with a catalytic quantity of dimethylaminopyridine (DMAP) (10 mg, 0.08 mmol, 0.2 eq.) was added. The mixture was subsequently heated at 50°C under argon. The DMF was azeotroped after 18h with heptane to give a white-yellow solid, which was dissolved in acidic water. The acidic aqueous solution was alkalinized to pH 4–5 with dropwise addition of NaOH 4M. The white precipitate that formed was filtered and dried under vacuum to give compound **1** as a pure white powder 218 mg, 90%)^1^H NMR (400 MHz, *DMSO-d*_*6*_) δ ppm 2.22 (s, 3H), 2.38 (s, 3H), 2.42 to 2.48 (m, 4H), 2.57 (t, J = 5.7 Hz, 2H), 3.21 to 3.38 (m, 4H), 3.43 to 3.56 (m, 4H), 4.14 (t, J = 5.7 Hz, 2H), 6.03 (s, 1H), 7.19 to 7.31 (m, 2H), 7.38 (d, J = 5.9 Hz, 1H), 8.20 (s, 1 H), 9.86 (s, 1 H), 11.46 (s, 1 H), 12.23 (bs, 1H).

Compound VII (AL776)

To a solution of **1** (218 mg, 0.37 mmol) and **AL621** [12] (171 mg, 1 eq) in dry DMF (3.6 mL) were added 1-ethyl-3-(3-dimethylaminopropyl) carbodiimide (EDCI) (78 μL, 1.2 eq.), hydroxybenzotriazole (HOBt) (60 mg, 1.2 eq.) and 4-dimethylaminopyridine DMAP (4.5 mg, 0.1 eq.). A precipitate appeared within a few minutes and the resulting brown mixture was further stirred at room temperature for 18h under argon. The DMF was azeotroped with heptane to give a crude solid, which was triturated in water, after which the resulting precipitate was filtered and dried. The resulting solid (440 mg) was purified by silica gel chromatography column (CH_2_Cl_2_/MeOH 9/1). Further purification by preparative thin layer chromatography (TLC) (silica plate, CH_2_Cl_2_/MeOH 9/1, two successive elutions) gave **VII** (AL776) as a pure white powder (136 mg, 36%).

^1^H NMR (300 MHz, *DMSO-d*_*6*_) δ ppm 2.19 (s, 3H), 2.21 (s, 3H), 2.37 (s, 3H), 2.41 to 2.47 (m, 4H), 2.52 to 2.64 (m, 8H), 3.42 to 3.53 (m, 4H), 3.59 (s, 2H), 4.05 to 4.20 (m, 4H), 6.01 (s, 1H), 7.14 (d, J = 9.4 Hz, 1H), 7.19 to 7.31 (m, 2H), 7.34 to 7.53 (m, 4H), 7.77 to 7.89 (m, 2H), 7.94 to 8.11 (m, 4H), 8.20 (s, 1 H), 8.59 (s, 1H), 8.90 (s, 1H), 9.86 (s, 1 H), 9.95 (s, 1H), 10.59 (s, 1 H), 10.58 (s, 1H), ESI *m/z* 1031.29 (MNa^+^ with ^35^Cl, ^35^Cl). Anal. (C_51_H_52_Cl_2_N_12_O_6_S) C, H, N.

### Cell culture

The cell lines used in the *in vitro* studies of AL776 included the mouse fibroblast NIH3T3 panel consisting of wild type (NIH3T3-WT) and cells transfected with EGFR (NIH3T3-Her14), the mouse mammary tumour cell line 4T1 as well as the triple negative human breast cancer cell line MDA-MB-231. The NIH3T3 wild type and Her14 (EGFR transfected) cells were a generous gift from Dr. Moulay Alaoui-Jamali (Lady Davis Institute for Medical Research Sir Mortimer B. Davis, Jewish General Hospital, Montreal, Canada). MDA-MB-231 human breast cancer cell line was purchased from American Type Culture Collection (ATCC, Manassas, VA, USA). 4T1 cells were a generous gift from Dr. Thierry Muanza (Department of Oncology, Division of Radiation Oncology, Jewish General Hospital, Montreal, Canada), originally isolated by Dr. Fred Miller (Karmanos Cancer Institute, MI, USA) [46]. All cell lines were maintained in Dulbecco Modified Eagle’s Medium (DMEM) supplemented with 10% FBS, 10 mM HEPES, 2 mM L-glutamine, gentamycin sulfate and fungizone (all reagents purchased from Wisent Inc., St-Bruno, Canada) and were grown in a humidified incubator with 5% carbon dioxide at 37°C.

### Drug Treatment

The K1-K2 molecules including AL776 were synthesized in our laboratory. Iressa (gefitinib, AstraZeneca) and dasatinib (Sprycel) were purchased from the Royal Victoria Hospital pharmacy (Montreal, Canada) and extracted from pills in our laboratory. All drugs were dissolved in DMSO to obtain a concentration of 10 mM (or lower). Drug dilutions were carried out under sterile conditions using DMEM (10% FBS or serum-free) medium and the final concentration of DMSO never exceeded 1% (v/v).

### Kinetic analysis of AL776 *in vitro* and *in vivo*

(A) Absorption kinetics analysis in NIH3T3-Her14 (EGFR) cells

NIH3T3-Her14 cells were seeded in 6-well plates (1 x 10^6^ cells/well) and grown in DMEM with 10% FBS for 24 h. Cells were then treated with 25 μM of AL776 and incubated at 37°C for 1h, 2h, 6h, 24h or 48h. The media was collected and extracted using twice the volume of methanol, on ice and centrifuged for 20 min at 13,000 rpm at 4°C. The resulting supernatant was filtered, evaporated to dryness and reconstituted with 100 μL of methanol. Cells were collected using trypsin and the cell pellet obtained was rinsed once with PBS and lysed with methanol (500 μL /tube) on ice for 1h. The lysate was centrifuged at 13,000 rpm for 20 min at 4°C and the resulting supernatant was collected into a separate tube and the remaining pellet was subjected to lysis two more times (250 μL /tube). The supernatant from each lysis step were pooled all together, filtered, evaporated to dryness and resuspended in 100 μL of methanol before further analyzing the samples. HPLC analysis was performed using a ACE 5 C18 5 μm column (150 mmx4.6 mm) under elution conditions of gradient 70–75% methanol:water for the first 5 min and isocratic methanol elution until 30 min, using a 0.75 mL/min flow rate. Analyses were performed using a Thermoquest P4000 equipped with a UV2000 detector and an AS300 Autosampler.

(B) Liquid chromatography-mass spectrometry (LC-MS) analysis of the hydrolysis of AL776 in vivo

CD-1 mice were divided into groups of three and treated with 80 mg/kg of AL776, injected intraperitoneally (i.p) or intravenously (i.v). Mice were sacrificed and their plasma was collected for further analysis after 0, 5, 15 and 30 min after drug treatment. Plasma samples were extracted using twice the volume of methanol and lysed at 13,000 rpm for 30 min at 4°C. The resulting supernatant from each sample was collected, filtered and evaporated to dryness before reconstituting in 100 μL of methanol. LC-MS analyses were performed on a Synapt G2-S instrument coupled with an Acquity UPLC Class I system both from Waters. Elution rate was set at 500 μL/min using an Eclipse XDB C8, 3.5 um, 2.1 x 100 mm chromatographic column from Agilent Technologies. The experiments were performed with the following eluents: 0.1% aqueous formic acid (eluent A) and methanol (eluent B). The initial mobile phase was 30% B, which was maintained for 0.2 min at the beginning of the run. The following gradient elution was subsequently applied: 30 to 50% B from 0.2 to 4 min; 50 to 80% B from 4 to 10 min; held at 80% B from 10 to 11 min. Thereafter, eluent B was decreased to 30% in 0.2 min and held constant for up to 15 min to allow the column to equilibrate. Each sample was diluted with methanol in order to avoid detector saturation and 3 L aliquots of the resulting solutions were injected. Analyses were performed with the electrospray interface in positive ion mode and mass spectra acquired from m/z 100 to 1200. For accurate mass determination, Leucine Enkephalin was used as lock mass. The MassLynx software was used for instrument control, data acquisition and data processing.

### *In vitro* kinase assay

The EGFR and c-Src kinase assays were performed in 96-well plates (NuncMaxisorp) coated with PGT (poly L-glutamic acid L-tyrosine, 4:1, Sigma Aldrich, MO, USA) and incubated at 37°C for 48h prior to using. PGT served as the substrate to be phosphorylated by EGFR (Enzo Life Sciences Inc, NY, USA, Signal Chem, Richmond, Canada) and c-Src (Enzo Life Sciences Inc, NY, USA, Signal Chem, Richmond, Canada) in the presence of ATP (50 μM). A dose range of drugs (**I-VII**, gefitinib or dasatinib) was added to compete with ATP to bind and inhibit the ATP-binding site in the kinase domain of EGFR or c-Src. To each well, 15 ng of EGFR (20 μg/ml) or 6 ng of c-Src (0.1 μg/μl) were added. The phosphorylated substrate was detected using an HRP-conjugated anti-phosphotyrosine antibody (Santa Cruz Biotechnology, CA). The signal was developed by the addition of 3, 3’, 5, 5’-tetramethylbenzidine peroxidase substrate (Kierkegaard and Perry Laboratories, Gaithersburg, MD) and the colorimetric reaction was monitored at 450 nm using a microplate reader ELx808 (BioTek Instruments). The IC_50_ values were calculated using GraphPad Prism 6.0 (GraphPadSoftware, Inc., San Diego, CA). Each experiment was carried out at least three times, in duplicate.

### Molecular Modeling

AL776 was modeled in the EGFR kinase pocket using the Protein Data Bank (PDB) structure [26] with code 1M17 downloaded from www.rcsb.org. The quinazoline portion of bound erlotinib in 1M17 was used as a template to construct and minimize a bound pose of AL776. Minimizations were carried out in the MOE 2013.08 [47] software using the Amber10:EHT forcefield with R-Field electrostatics. AL776 was also modeled in the c-Src kinase pocket using the PDB structure 3G5D [27]. AL776 was constructed and minimized in 3G5D staring with the bound dasatinib ligand as the template. The modeling was carried out in the MOE 2013.08 software using the Amber10:EHT forcefield and R-Field electrostatics for minimizations.

### Growth inhibition assay

Growth inhibition was measured in cells using the sulforhodamine B (SRB) assay [48]. NIH3T3 (wildtype and EGFR transfected), MDA-MB-231 and 4T1 cell lines were grown in 10% FBS containing media (DMEM) were plated (2000–5000 cells/well) in 96-well plates and allowed to attach overnight (37°C, 5% CO_2_). Twenty-four hours later, they were treated with a dose range of drugs (**I-VII**, gefitinib and dasatinib) or media (for control) and allowed to grow in the incubator for the next 120h (5 days) at 37°C. At the end of this 5-day treatment period, they were fixed in 50% trichloroacetic acid (TCA) for 2–3h at 4°C, washed four times under cold tap water and stained with sulforhodamine B (0.4%) overnight at room temperature. The plates were subsequently rinsed with 1% acetic acid, and allowed to dry overnight. The stained cells were then dissolved using 10 mM Tris-Base and the plates were read using a microplate reader ELx808 at 492 nm. The results were analyzed using GraphPad Prism 6.0 (GraphPadSoftware, Inc., San Diego, CA) and the sigmoidal dose response curve was used to determine IC_50_ values. Each experiment was carried out at least four times, in triplicate.

### Wound-healing assay

MDA-MB-231 and 4T1 cells were plated in 6-well plates (500,000 cells/well) and allowed to attach overnight (37°C, 5% CO_2_). The following day, media was removed and a cross scratch was made in the middle of the cell monolayer. Cells were washed twice with PBS, and treated with 100 nM of drugs (AL776, dasatinib, gefitinib) or just media (control) for a period of 24 hours. The scratch was visualized at two different time points (0 and 24h) using the Leica DM IL inverted microscope (10X) and images were obtained using the Leica DFC300FX camera.

### Boyden chamber invasion assay

The invasive property of MDA-MB-231 and 4T1 cells was determined using the Boyden-chamber invasion assay. Cells suspended in serum-free media were plated (120–150,000 cells/well) onto polycarbonate transwell inserts (8 μm pore size, BD Biosciences) coated with matrigel (6%) (BD Biosciences), separating the top and bottom chambers (50 μl/filter). The cells were allowed to attach for a few hours and subsequently, serum-free media with or without drugs (AL776, dasatinib, gefitinib) was added to the top chambers of the inserts, whereas 10% FBS containing complete media with or without drugs was added to the bottom chambers creating a chemo-attractive gradient. The invasive property of cells was observed 24h after treatment by fixing cells in formalin and staining with 0.1% crystal violet (Sigma-Aldrich Canada Ltd). Cells attached on the upper surface of the insert (top chamber) were removed by gently scraping away with a cotton swab, whereas those that invaded onto the lower side of the insert were observed using a microscope (Leica DM IL inverted microscope, 10X). Images of five non-overlapping fields of invading cells were captured using the Leica DFC300FX camera and quantification was done using the Scion Image Analysis (3.53.0.0) software and ImageJ 1.46r software. The results were expressed in terms of percentage of invading cells relative to control and the bar graphs represent the average of three independent experiments.

### Western blot

NIH3T3-Her14 and 4T1 cells grown in 10% FBS containing media were plated (~ 1 x 10^6^ cells/well) in 6-well plates and allowed to attach overnight (37°C, 5% CO_2_). The cells were rinsed twice with PBS twenty-four hours later and starved overnight on addition of serum-free media. Thereafter, they were treated with different doses of AL776 for 2 hours, washed with PBS (twice) and stimulated with 50 ng/ml EGF for 30 min at 37°C. Cells were washed, detached by scraping in ice-cold PBS and collected by centrifugation for 15 min at 3000 rpm. Cell pellets were re-suspended in cold lysis buffer 50 mM Tris-HCl pH 7.5; 150 mM NaCl; 1% Nonidet P-40, 1 mM EDTA; 5 mM NaF; 1 mM Na_3_VO_4_; protease inhibitor tablet (Roche Biochemicals, Laval, Canada)]. Lysates were kept on ice for 1 h and collected by centrifugation at 13,000 rpm for 20 min at 4°C. The concentration of protein was determined using the Bio-Rad protein assay kit (Bio-Rad laboratories, Hercules, CA). Equal amounts of proteins were loaded, resolved on 10% SDS-PAGE and thereafter transferred to a polyvinylidenedifluoride (PVDF) membrane (Milipore, Bedford, MA). Membranes were blocked with 5% milk in TBST (20 mM Tris-HCl, 137 mM NaCl, 0.1% Tween 20) overnight at 4°C followed by incubation with phosphotyrosine antibodies such as phospho-EGFR Y1068 (Cell Signaling Technology, USA, 1:4000) and phospho-Src Y416 (Cell Signaling Technology, USA, 1:1000) in 5% milk, at 4°C overnight. The membranes were washed with TBST and incubated with respective secondary antibodies for 1h at room temperature in 5% blocking solution. After incubation with antibodies against phosphotyrosines, the membranes were stripped using the Restore Stripping buffer (Thermo Scientific, Rockford, IL, United States) and probed for total EGFR (Santa Cruz, CA, USA) and total Src (Cell Signaling Technology, USA) antibodies along with GAPDH or beta-actin (Santa Cruz, CA, USA) antibodies. Immunoblot bands were visualized using ECL kit and enhanced chemiluminescence system (GE Healthcare).

### Characterization of apoptosis using flow cytometry

NIH3T3 cell lines (wild type and EGFR transfected) were plated in 6-well plates (500,000 cells/well) and allowed to grow overnight (37°C, 5% CO_2_). The next day, fresh media with or without different doses of drugs (AL776, dasatinib, gefitinib) were added to the cells and incubated for a period of 48 hours. They were subsequently collected using trypsin (Wisent Inc., St-Bruno, Canada), centrifuged (2000 rpm, 5 min), rinsed with PBS and centrifuged again to obtain a cell pellet. The pellet was re-suspended in binding buffer (1X) and prior to being analyzed, annexin V-FITC and PI stains (eBioscience, San Diego, CA, USA) were added to the cells and incubated for 15 min in the dark at 4°C. Annexin V–FITC and PI binding were analyzed with a Becton Dickinson FACScan. Data were collected using logarithmic amplification of both the FL1 (FITC) and FL2 (PI) channels. Quadrant analysis of coordinate dot plots was done with CellQuestPro software. The experiment was carried out three times.

### *In vivo* efficacy and pharmacodynamics

*In vivo* efficacy and pharmacodynamic studies were carried out in female Balb/c mice (Charles River Laboratories, USA) strictly in accordance with the protocol (#4934) approved by the Facility Animal Care Committee (FACC), McGill University. Mice were implanted with 4T1 mammary tumour cells, 15 million cells/flank (suspended in 200 μL PBS), subcutaneously. For the efficacy study, once the tumours reached an average size of 80 mm^3^, the mice were randomized into groups of 6 (n = 6) and treated (intravenously, i.v, once/day) with vehicle (20% + cremophor + 20% ethanol + 60% saline) or 40 mg/kg of AL776. Tumour growth was monitored for the next two weeks by measuring tumour volumes using the formula [4/3*3.14*L/2*(W/2)^2] alternate days, along with the body weight.

For the pharmacodynamic study, female Balb/c mice bearing 4T1 tumours were divided into groups of four and treated with vehicle (20% cremophor + 20% ethanol + 60% saline), AL776 (40 mg/kg) or combination of gefitinib + dasatinib (20mg/kg each). Mice were sacrificed 1h or 24h after treatment and their tumours were collected and snap frozen at -80°C for further analysis using western blots. Tumours were crushed using a mortar/pestle chilled using liquid nitrogen and lysed with RIPA buffer diluted to a 1X concentration and supplemented with 1mM PMSF (Cell Signaling Technology, USA). Western blot analysis was carried out with the tumour samples, similar to the method described earlier.

### Statistical Significance

Statistical significance for *in vitro* assays was carried out using the unpaired, two-tailed student t-test with p < 0.05 indicating significance. For the *in vivo* pharmacodynamic experiments, statistical significance was determined using multiple t-test (Holm-Sidak method, with alpha = 5.0%). Each row was analyzed individually, without assuming a consistent SD. P value < 0.05 was considered statistically significant. GraphPad Prism 6.0 (GraphPadSoftware, Inc., San Diego, CA) was used for statistical analysis.

## Supporting Information

S1 Fig High performance liquid chromatography (HPLC) analysis of the hydrolysis of AL776 inside the cells. The intracellular hydrolysis of AL776 was studied by treating NIH3T3-Her14 (EGFR transfected) cells with 25 μM of AL776 for 1, 2, 6, 24 and 48h. The HPLC spectra show the slow internalization and degradation products (AL621 and dasatinib) obtained from AL776 hydrolysis mediated by intracellular esterases.
(TIF)

S2 Fig Growth inhibition in NIH3T3 wild type and Her14 (EGFR-transfected) cell lines. The growth inhibitory property of each EGFR-c-Src targeting molecule in the series was determined using the sulforhodamine B (SRB) assay. Gefitinib and dasatinib were used as control drugs for comparison, and the IC_50_ values for growth inhibition were determined using the GraphPad Prism 6.0 software.
(TIF)

S3 Fig Toxicity of AL776 *in vivo*. The toxicity of (**A**) AL776 (40 mg/kg) and the combination of (**B**) gefitinib + dasatinib (20 mg/kg each) was determined in CD-1 mice (n = 3) treated with the drug (intravenous, i.v.) for 4 consecutive days. The effect of toxicity was determined by monitoring the average body weight (g) of each treated group compared with the vehicle control. Greater than 15% weight loss was considered toxic.
(TIF)

S4 Fig Pharmacodynamics of AL776. Female Balb/c mice (n = 4) with 4T1 mammary tumours were treated with (**A**) 40 mg/kg of AL776 or the combination of (**B**) gefitinib + dasatinib (20 mg/kg each) compared with the vehicle control. Mice were sacrificed 1h or 24h after drug exposure and the tumours were collected, processed and inhibition of phosphorylated proteins (EGFR, c-Src, ERK1/2) was assessed using western blots.
(TIF)
