# Supplementary figures and images for: Target Modulation by a Kinase Inhibitor Engineered to Induce a Tandem Blockade of the Epidermal Growth Factor Receptor (EGFR) and c-Src: The Concept of Type III Combi-Targeting
Source: PLoS One. 2015 Feb 6;10(2):e0117215. doi: 10.1371/journal.pone.0117215 (PMC4414309; doi:10.1371/journal.pone.0117215)

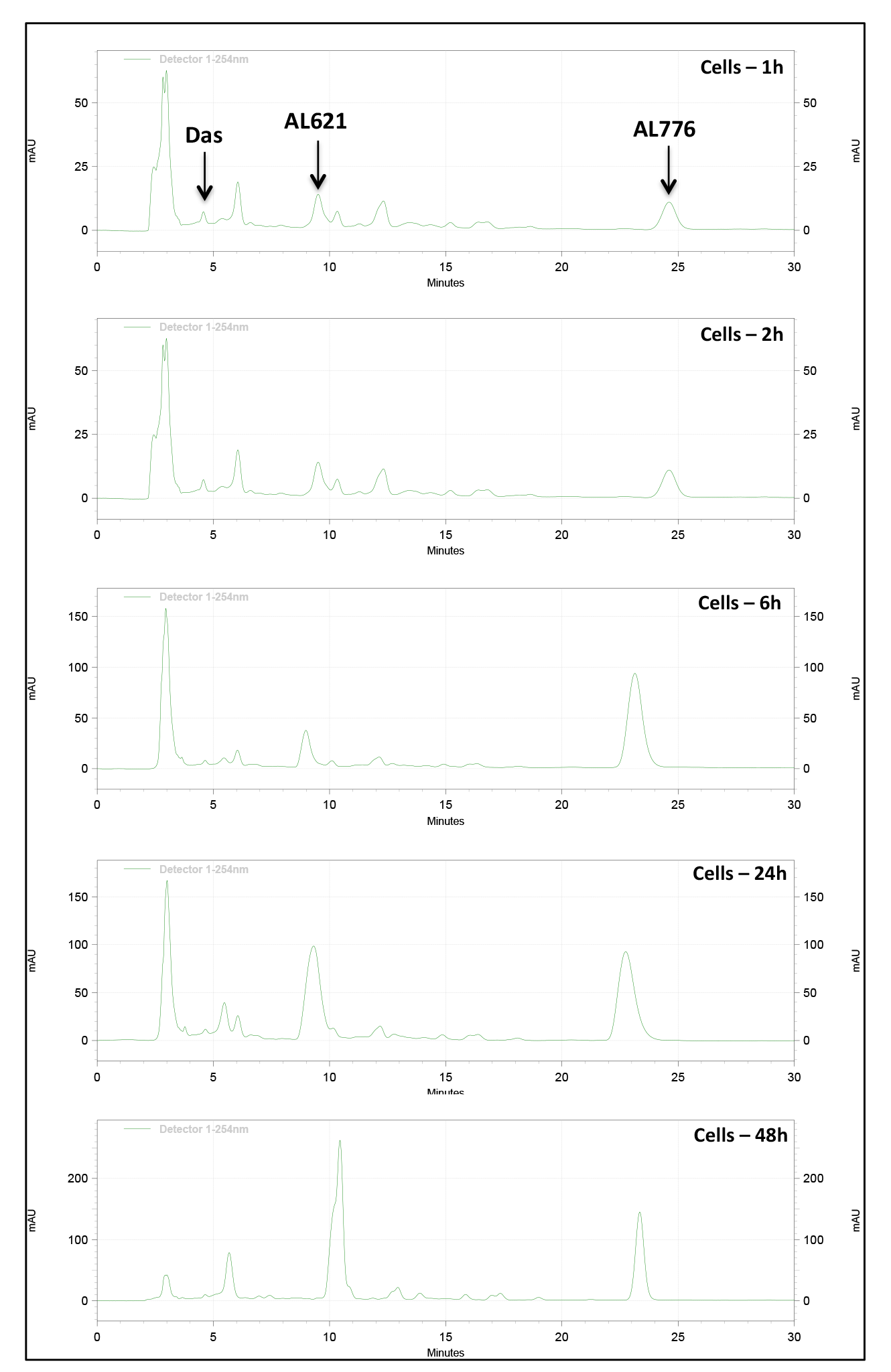

Supplement: S1 Fig — The intracellular hydrolysis of AL776 was studied by treating NIH3T3-Her14 (EGFR transfected) cells with 25 μM of AL776 for 1, 2, 6, 24 and 48h. The HPLC spectra show the slow internalization and degradation products (AL621 and dasatinib) obtained from AL776 hydrolysis mediated by intracellular esterases. (TIF) [file pone.0117215.s001.tif]

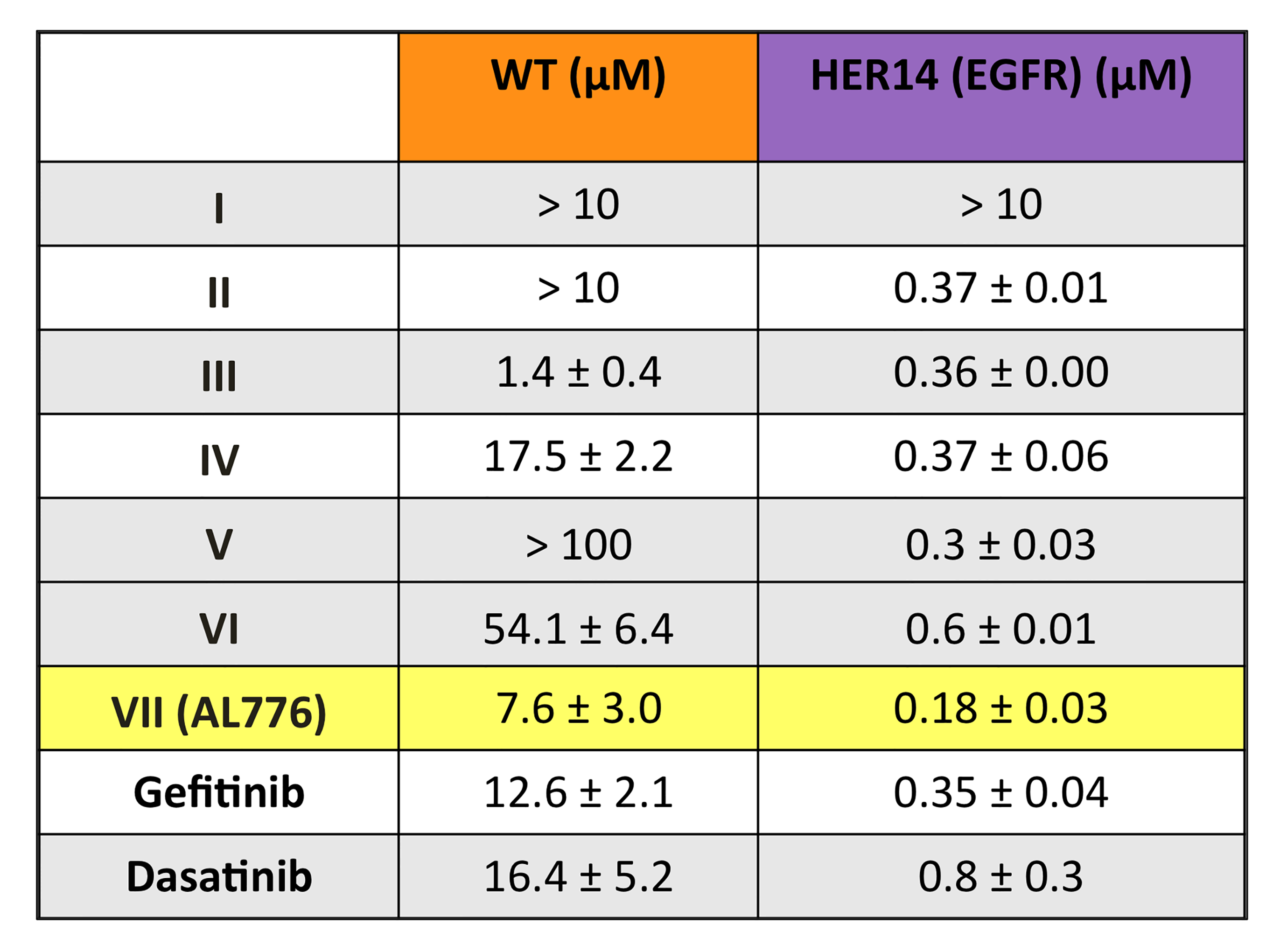

Supplement: S2 Fig — The growth inhibitory property of each EGFR-c-Src targeting molecule in the series was determined using the sulforhodamine B (SRB) assay. Gefitinib and dasatinib were used as control drugs for comparison, and the IC50 values for growth inhibition were determined using the GraphPad Prism 6.0 software. (TIF) [file pone.0117215.s002.tif]

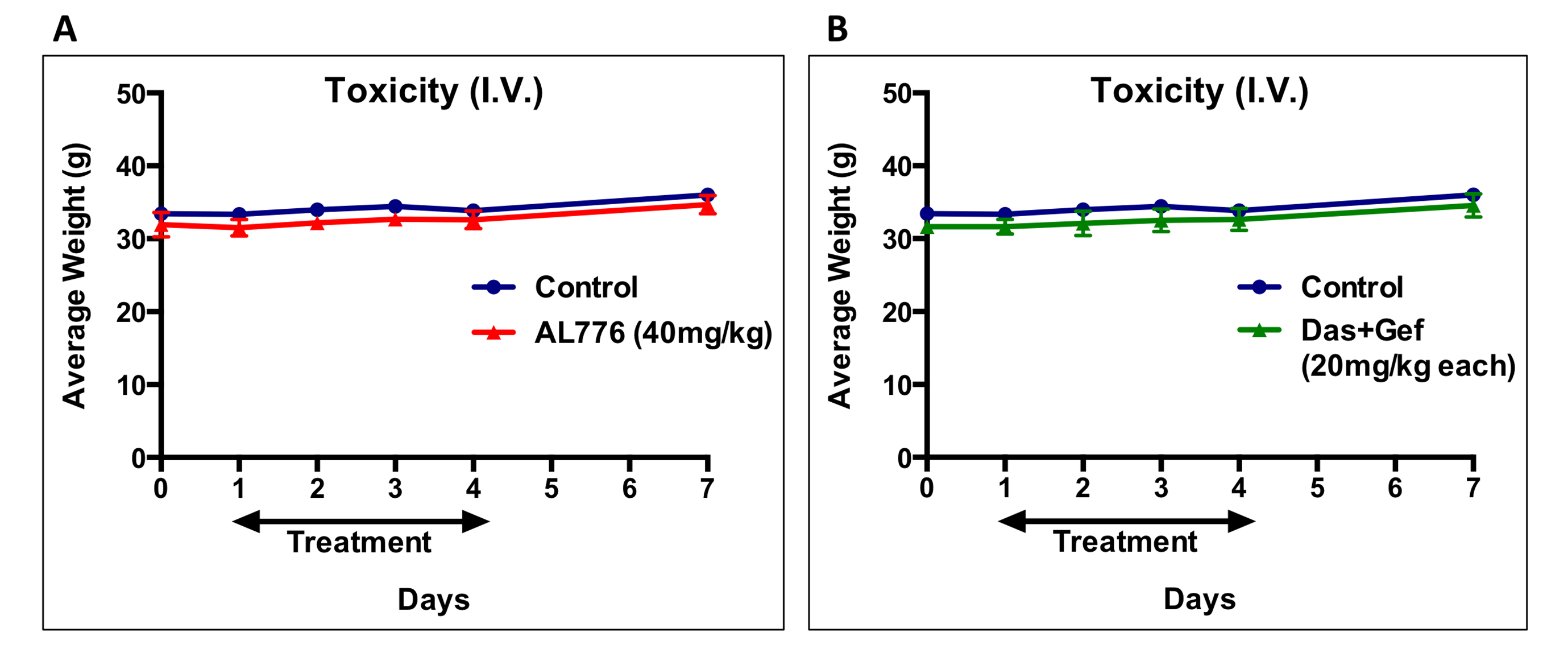

Supplement: S3 Fig — The toxicity of (A) AL776 (40 mg/kg) and the combination of (B) gefitinib + dasatinib (20 mg/kg each) was determined in CD-1 mice (n = 3) treated with the drug (intravenous, i.v.) for 4 consecutive days. The effect of toxicity was determined by monitoring the average body weight (g) of each treated group compared with the vehicle control. Greater than 15% weight loss was considered toxic. (TIF) [file pone.0117215.s003.tif]

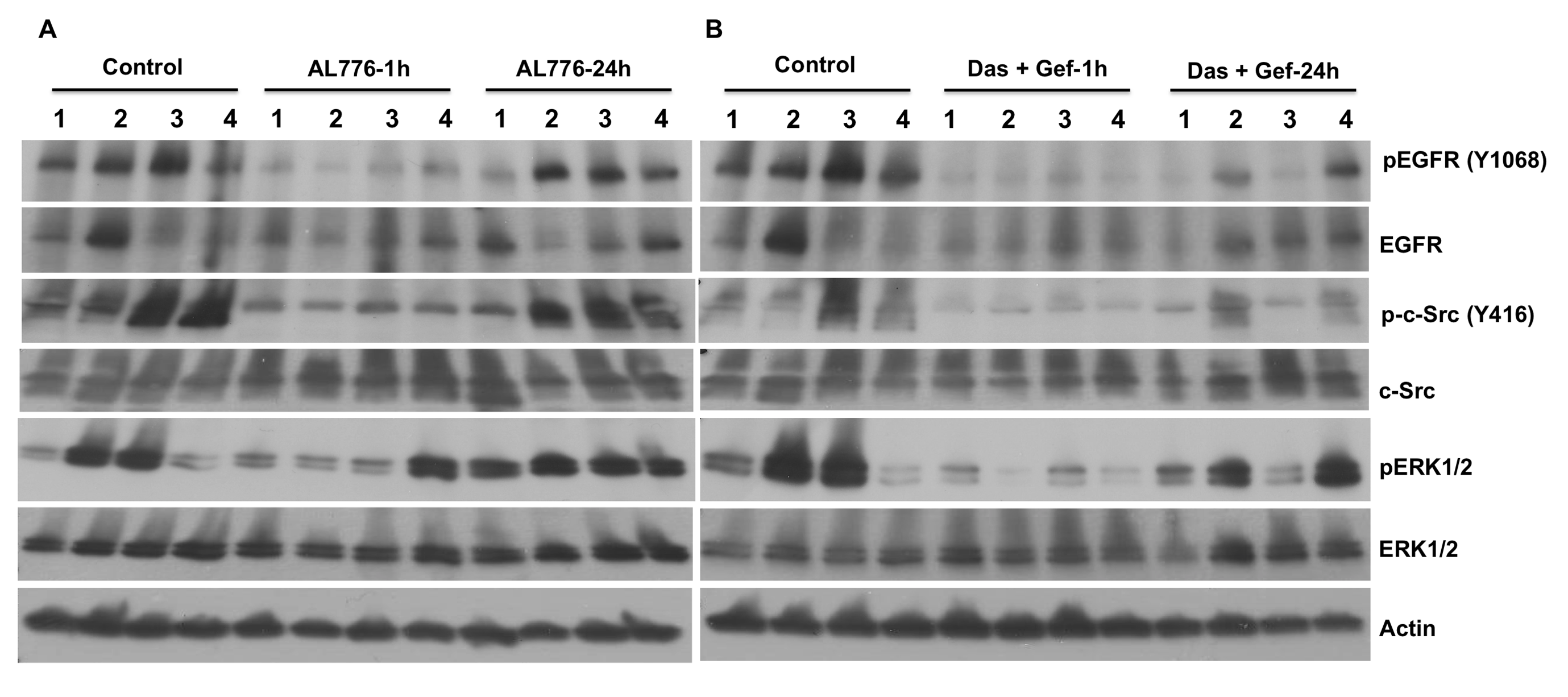

Supplement: S4 Fig — Female Balb/c mice (n = 4) with 4T1 mammary tumours were treated with (A) 40 mg/kg of AL776 or the combination of (B) gefitinib + dasatinib (20 mg/kg each) compared with the vehicle control. Mice were sacrificed 1h or 24h after drug exposure and the tumours were collected, processed and inhibition of phosphorylated proteins (EGFR, c-Src, ERK1/2) was assessed using western blots. (TIF) [file pone.0117215.s004.tif]
